# A review on the treatment of hyperlipidemia with Erchen Decoction

**DOI:** 10.3389/fphar.2024.1445950

**Published:** 2024-11-13

**Authors:** Xinyang Tian, Peiyu Liu, Ruolin Wang, Yawei Hou, Ying Zhou, Chunyan Wang, Guiju Zhang

**Affiliations:** ^1^ Institute of Traditional Chinese Medicine Literature and Culture, Shandong University of Traditional Chinese Medicine, Jinan, Shandong, China; ^2^ The Second Clinical College, Guangzhou University of Chinese Medicine, Guangzhou, Guangdong, China; ^3^ First Clinical Medical College, Shandong University of Traditional Chinese Medicine, Jinan, Shandong, China

**Keywords:** Traditional Chinese Medicine, Erchen Decoction, hyperlipidemia, atherosclerotic cardiovascular diseases, cardiovascular disease

## Abstract

Hyperlipidemia, commonly referred to as dyslipidemia, is characterized by elevated serum cholesterol and/or triglyceride levels. This condition contributes significantly to the high mortality rates associated with cardiovascular diseases, posing a serious threat to global health. Although statins remain the predominant pharmacological treatment for hyperlipidemia, their associated side effects have led to a growing interest in alternative therapeutic approaches. Traditional Chinese Medicine (TCM) is exploring these alternatives, with the Erchen Decoction (ECD) emerging as a promising candidate. This review aims to summarize current clinical research, elucidate the mechanisms of action, and assess the compatibility of ECD in the management of hyperlipidemia. By doing so, we hope to provide valuable insights and references for clinical practice and future research.

## 1 Introduction

Hyperlipidemia usually refers to elevated levels of total cholesterol (TC), triglycerides (TG), and/or low-density lipoprotein cholesterol (LDL-C) in the serum (Li et al., 2023a), along with a decrease in high-density lipoprotein cholesterol (HDL-C) (Luo et al., 2020). An abnormality in any of these indicators can be diagnosed as hyperlipidemia, also known as dyslipidemia. The prevalence of dyslipidemia in China is showing a year-on-year increase. According to research conducted in 2023, involving approximately 1.3 million Chinese participants, the prevalence of dyslipidemia was found to be 42.1% (Xia et al., 2023). This figure shows a significant rising trend compared to the total prevalence of dyslipidemia among Chinese adults in 2018 (35.6%) (Li et al., 2023a). Hyperlipidemia is closely associated with atherosclerotic cardiovascular diseases (ASCVD), and evidence from genetic, epidemiological, and clinical studies indicates that elevated LDL-C is a major factor in the pathogenesis and mortality of ASCVD (Ference et al., 2017). LDL-C deposits in arterial walls, where it binds with intimal proteoglycans, leading to gradual pathological changes and accumulation at sites prone to plaque formation (Ference et al., 2017; Libby et al., 2019). LDL-C plays a critical role in the development of atherosclerotic plaques and subsequent cardiovascular events (Mach et al., 2020). Cardiovascular diseases (CVDs), primarily resulting from ASCVD, are the leading cause of death among urban and rural populations in China, accounting for over 40% of all mortalities (Li et al., 2023b). Although the mortality rate due to cardiovascular diseases (CVD) has declined in Europe, it remains the most common cause of death among the population (Timmis et al., 2024). Other risk factors associated with hyperlipidemia, such as diabetes mellitus, hypertension (Su and Yu, 2009), and non-alcoholic fatty liver disease (NAFLD) (Deng et al., 2023), also pose significant health threats. Statins are commonly used to treat hyperlipidemia, effectively inhibit the condition, and reduce LDL-C levels (Karalis, 2021). However, long-term excessive use of statins can lead to adverse effects, such as myopathy, liver injury, severe renal impairment, and cytotoxicity in both humans and animals *in vitro* (Liu et al., 2019). Therefore, there is an urgent need to identify medications that are both highly effective and have fewer side effects.

**FIGURE 1:**
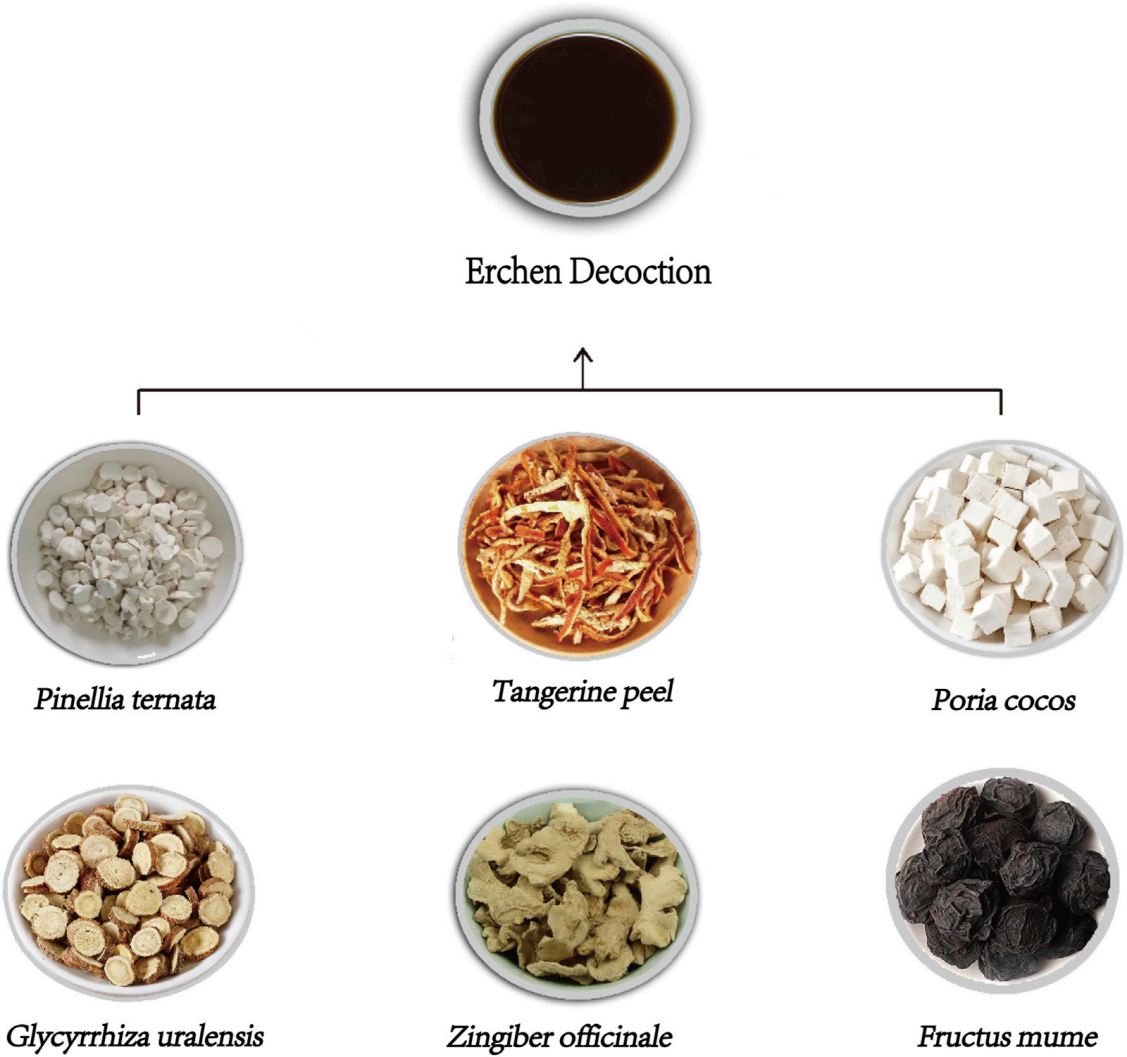
Composition of erchen decoction.

ECD is a classic prescription in TCM, originating from the “Prescriptions of the Bureau of Taiping People’s Welfare Pharmacy.” This formulation is effective for drying dampness, eliminating phlegm, regulating Qi, and harmonizing the middle, making it suitable for treating the phlegm-dampness syndrome. The formulation consisted of *Pinellia ternata* [Araceae], *Tangerine peel* [Rutaceae], *Poria cocos* [Polyporaceae], *Glycyrrhiza uralensis* [Fabaceae], *Zingiber officinale* [Zingiberaceae], and *Fructus mume* [Rosaceae] (Table 1; Figure 1). Pinellia ternate (P. ternata) and Tangerine peel (T.peel) work to dry dampness and eliminate phlegm, Whereas Poria cocos (P.cocos) dries dampness and regulates Qi. Glycyrrhiza uralensis (G.uralensis) harmonizes various metabolites, strengthens the spleen, and enhances the middle Jiao. ECD and its modified versions are widely used to treat the lung system, heart system, and metabolic disorders (Xiang, 2023). Hyperlipidemia, a common metabolic disease, is frequently treated with ECD. Current research has primarily focused on four aspects: clinical studies, molecular mechanisms, pharmacological effects, and chemical metabolites. This article systematically reviews the research on the treatment of hyperlipidemia with ECD, aiming to provide a comprehensive overview of the forefront of research on this prescription and offer effective references for establishing further research directions for ECD.

**TABLE 1:** Composition of botanical drugs in ECD.

Scientific name	Family	Common name (traditional name)	Part(s) of drug used
*Pinellia ternata* (Thunb.) Breit	Araceae	Pinellia ternata	“Dried tuber
*Citrus reticulata Blanco*	Rutaceae	Tangerine Peel (chenpi)	Dried fruit peel
*Poria cocos* (Schw.) Wolf	Polyporaceae	Poria cocos	Dried sclerotium
*Glycyrrhiza uralensis* Fisch	Fabaceae	Glycyrrhiza uralensis	Dried root
*Zingiber officinale* (Willd.) Rosc	Zingiberaceae	Zingiber officinale	Dried rhizome
*Prunus mume* (Sieb.) Sieb. et zucc	Rosecea	Fructus mume	Dried fruit

## 2 Clinical application of Erchen Decoction in treating hyperlipidemia

### 2.1 Case analysis of Erchen Decoction in the treatment of hyperlipidemia

ECD has demonstrated significant efficacy in the treatment of hyperlipidemia, often involving modifications, adaptations, or combinations based on its formulation. Wang et al. suggested that the accumulation of phlegm-dampness in the body slows the circulation and dispersion of body fluids, leading to the stagnation of subtle substances and resulting in hyperlipidemia. Given the close relationship between hyperlipidemia and phlegm generated by the spleen and kidney, treatments should focus on addressing phlegm-dampness. Their study utilized ECD alongside Modified Shenling Baizhu Powder, incorporating lotus leaf and gynostemma, which resulted in significant symptom relief after four treatment courses (Zhu and Wang, 2022). Kuang Bin emphasized the importance of eliminating phlegm and dampness in hyperlipidemia treatment. In their study involving 88 patients, modifications to ECD included the addition of botanical drugs effective in relieving water and expelling dampness, such as Coix seed, Rhizoma Alismatis, and Polyporus umbellatus. This protocol achieved an overall effectiveness rate of 87.5% and successfully controlled lipid levels in patients (Kuang and Huang, 2017). Wang et al. reported an increased prevalence of phlegm-turbidity obstructive hyperlipidemia among patients. They employed a treatment regimen combining Modified ECD with Jiaosanxian, effectively addressing both the underlying causes and symptoms. Following a 12-week treatment period, the results demonstrated a significant improvement, surpassing the efficacy of pravastatin and leading to notable reductions in serum levels of TC and LDL-C in the patients. (Wang, 2022). Luo et al. proposed that the pathogenesis of hyperlipidemia primarily involves organ deficiency, particularly the spleen, with phlegm-turbidity and blood stasis as secondary manifestations. Thus, their treatment focused on invigorating the liver and strengthening the spleen in conjunction with dampness removed. ECD and Modified Astragali Radix Jianzhong Decoction were prescribed, which resulted in the disappearance of symptoms after 14 doses (Luo, 2014). Li et al. highlighted the crucial roles of the spleen and stomach in metabolism. Weakness in these organs often leads to phlegm turbidity and blood stasis, which persistently contribute to hyperlipidemia. To address this, treatment commenced by targeting spleen qi deficiency syndrome, recognizing spleen qi deficiency as the root cause and blood stasis as the manifestation. Modifications to ECD, Sijunzi Decoction, and Semen Persicae Siwu Decoction based on symptoms resulted in high patient satisfaction (Li and Liu, 2017).

### 2.2 Clinical observation study on the treatment of hyperlipidemia with Erchen Decoction

Clinical research indicates that integrating additional medicinal botanical drugs into ECD, or using ECD in combination with other formulas, can enhance the precision of treatment for specific hyperlipidemia patterns (Table 2). Luo et al. conducted a study involving 115 patients with hyperlipidemia, and randomly assigned them to either a treatment or control group. The treatment group received Modified ECD, with patients classified into syndrome types of qi stagnation and blood stasis, liver yang hyperactivity, and liver and kidney yin deficiency, and received tailored botanical drug treatments accordingly. After three treatment courses, a continuous observation period of 3 weeks revealed that the treatment group exhibited higher TC levels than the control group, although lower than their pre-treatment levels. The TG levels in the treatment group were lower than those in the control group. The lipid-lowering effects were statistically significant in both groups, indicating that different modifications of ECD can effectively address hyperlipidemia (Luo, 2012).

**TABLE 2:** Clinical application of Erchen Decoction in the treatment of hyperlipidemia.

	Group	Medication (dosage of drug)	Number of patients	Age (average age)	Treatment effect	The medication time	Inclusion time	Site	References
1	Observation group	On the basis of the control group, Tangerine Peel, Pinellia ternata, poria cocos, burnt Crataegus pinnatifida (10g, 10g, 20g, 20g), Zingiber officinale (3 slices) were added	58	55.6 ± 5.5 (N)	The marked effectiveness rate was 41.4%, and the overall effectiveness rate was 89.7%	3 times a day, 1 dose in the morning, 1 dose at noon and 1 dose in the evening, 30 days was one course, and treatment last 3 consecutive courses	May 2008-September 2011	Guizhou Bijie College Hospital	Luo (2012)
	Control group	Gefilozil Ccapsules	57	54.1 ± 5.7 (N)	The marked effectiveness rate was 43.9%, and the overall effectiveness rate was 91.2%	Twice a day, 30 days was one course, and treatment last 3 consecutive courses	May 2008-September 2011	Guizhou Bijie College Hospital	Luo (2012)
2	Observation group	burnt Crataegus pinnatifida, tangerine peel, Pinellia ternata, radix bupleuri, Largehead Atractylodes Rhizome, Astragalus mongholicus, poria cocos, Rhizoma Alismatis, safflower, salvia miltiorrhiza, l Glycyrrhiza uralensis (30g, 12g, 9g, 9g, 9g, 9g, 9g, 12g, 9g, 10g, 10g, 3g)	47	48.3 ± 12.4	The marked effectiveness rate was 69.1%, and the efficacy rate was 85.1%	Once a day, the treatment time was 12 weeks	February 2007-October 2012	Anhui Huaibei Hospital of Traditional Chinese Medicine	Ding (2013)
	Control group	Rosuvastain	47	50.6 ± 11.2	The marked effectiveness rate was 63.9%, and the efficacy rate was 83.0%	1 dose in the morning and 1 dose in the evening, the treatment time was 12 weeks	February 2007-October 2012	Anhui Huaibei Hospital of Traditional Chinese Medicine	Ding (2013)
3	Observation group	Poria cocos, Medicated Leaven, Hawthorn, Codonopsis pilosula, Pinellia ternata, Tangerine peel, Atractylodes Salvia miltiorrhiza, baked Glycyrrhiza uralensis (15g, 15g, 15g, 15g, 10g, 10g, 10g, 10g, 6g)	40	50.45 ± 6.37	The adverse reaction rate was 7.5%	Twice a day, the treatment time was 2 weeks	January 2019-December 2020	Jiangxi Provincial Hospital of Traditional Chinese Medicine	Chen et al. (2021)
	Control group	Atorvastatin	40	50.27 ± 6.44	The adverse reaction rate was 5.0%	Once a day, the treatment time was 2 weeks	January 2019-December 2020 January 2020-June 2021	Jiangxi Provincial Hospital of Traditional Chinese Medicine	Chen et al. (2021)
4	Observation group	Pinellia ternata, tangerine peel, poria cocos, polygonum cuspidatum, Rhizoma Alismatis, lotus leaf, raw hawthorn, stir-fried cassia seed, Salvia miltiorrhiza, baked Glycyrrhiza uralensis (15g, 9g, 15g, 15g, 9g, 15g, 15g, 12g, 15g, 6g)	39	58.82 ± 9.87	The efficacy rate was 97.43%	Once a day, the treatment time was 12 weeks	January 2020-June 2021	Yueyang Hospital of Integrated Traditional Chinese and Western Medicine	Yin and Fu (2022a)
	Control group	Atorvastatin	39	58.90 ± 9.92	The efficacy rate was 82.62%	2 bags per person per day, the treatment time was 12 weeks	January 2020-June 2021	Yueyang Hospital of Integrated Traditional Chinese and Western Medicine	Yin and Fu (2022a)

Ding conducted a clinical controlled trial of 94 patients with hyperlipidemia. The control group received rosuvastatin and acipimox capsules, while the treatment group was administered doctor-formulated Crataegus pinnatifida ECD. Post-treatment, the efficacy rate was 85.1% in the treatment group compared to 83.0% in the control group, with no significant differences between the two, suggesting that Crataegus pinnatifida ECD has clinical efficacy comparable to that of conventional lipid-lowering medications (Ding, 2013). The study conducted by Chen et al. randomly assigned 80 patients with hyperlipidemia into two groups, the control group receiving atorvastatin, and the observation group receiving additional modified ECD combined with spleen-strengthening turbidity-clearing moxibustion (Chen et al., 2021). Both groups were treated continuously for 14 days, showing improvements in TC, TG, HDL-C, and LDL-C levels compared to pre-treatment values, with the observation group demonstrating superior outcomes. Yin and Fu employed modified ECD alongside atorvastatin to treat phlegm-damp obstructive hyperlipidemia, effectively regulating lipid metabolism and stabilizing abnormal lipid levels in patients (Yin and Fu, 2022a).

### 2.3 Erchen Decoction in the treatment of cardiovascular diseases caused by hyperlipidemia

As previously mentioned, when blood cholesterol levels exceed the body’s requirements, LDL-C accumulates on arterial walls and bind with intimal proteoglycans to form aggregates that infiltrate the smooth muscle cells. Pathological changes occur when both smooth muscle and macrophages become laden with lipids (Ference et al., 2017), potentially leading to CVD if not addressed promptly. Dong et al. conducted a randomized controlled trial of 96 patients with coronary heart disease complicated by hyperlipidemia. The observation group received standard statin therapy in conjunction with ECD. The results indicated that the levels of TC, TG, and LDL-C decreased in both groups compared to pre-treatment levels, with the observation group exhibiting significantly lower levels than the control group. Additionally, HDL-C levels increased compared to pre-treatment values, with higher levels observed in the observation group. These findings suggest that combining ECD with statin therapy can effectively regulate lipid levels and inhibit inflammatory responses, significantly enhancing the treatment efficacy for coronary heart disease associated with hyperlipidemia (Dong et al., 2021).

Yin and Fu applied a Modified ECD combined with atorvastatin to treat phlegm-damp obstructive hyperlipidemia and assessed its effects on inflammatory markers, carotid artery plaques, and uric acid levels in patients. The results demonstrated significant reductions in serum interleukin-6 (IL-6), tumor necrosis factor alpha (TNF-α), and C-reactive protein (CRP) levels in both groups after treatment, along with notable decreases in uric acid levels. Furthermore, the observation group exhibited significantly lower levels of inflammatory factors and uric acid than did the control group. After treatment, carotid plaque Smax in the observation group was significantly reduced compared to that in the control group, indicating that Modified ECD combined with atorvastatin could effectively lower inflammatory factors, carotid plaque area, and uric acid levels in patients with phlegm-damp obstructive hyperlipidemia (Yin and Fu, 2022b). Li et al. suggested that carotid artery stenosis (CAS) results from intermingling and stagnation of phlegm turbidity and blood stasis in neck vessels. They successfully employed a Modified ECD combined with Siwu Decoction to lower blood lipids and prevent, reduce, and eliminate carotid artery plaques. After 8 weeks of treatment, symptoms improved in the treatment group, and TC, TG, LDL-C, and HDL-C levels in serum all showed significant enhancement (Li J. et al., 2019). Another study by Bai and Huang addressed coronary atherosclerotic heart disease (CAD) by applying the Erchen Decoction in combination with Xuefu Zhuyu Decoction. The total effective rate for symptoms such as chest pain, chest tightness, palpitations, fatigue, and shortness of breath was significantly higher in the observation group than in the control group, effectively alleviating lipid levels in patients with coronary heart disease and angina pectoris (Bai and Huang, 2019).

## 3 The mechanism of Erchen Decoction in the treatment of hyperlipidemia

### 3.1 Erchen Decoction enhances lipid metabolism

Hyperlipidemia is characterized by lipid metabolism disorders, which primarily result from abnormal lipid transportation, wherein lipid transporters and their receptors play a critical role (Milhem et al., 2024). ECD has been widely reported (Luo et al., 2020; Ding et al., 2022) to improve lipid metabolism disorders. A study by Ding et al. indicated that, without intervening in the energy intake of high-fat diet (HFD) rats, the use of Erchen Decoction could increase HDL-C levels while decreasing TC, TG, and LDL-C levels in HFD rats. Furthermore, ECD improved lipid metabolism and reduced blood glucose and insulin resistance by regulating the expression of Cav-1, LDLR, and ABCA1 mRNA in HFD rats, as well as the level of SRB1 in visceral adipose tissue (Ding et al., 2018). Zhang et al. showed that after intervention with ECD, the body weight, Lee’s index, and abdominal circumference of mice significantly decreased, and the levels of TG and TC in serum also significantly declined. At the same time, PPARγ mRNA expression in visceral fat was significantly higher than that in the control group (Zhang M. et al., 2020). PPARγ is a key transcription factor in fat metabolism; activating PPARγ can reduce fatty acids transported to the liver and muscles, decrease fat synthesis, and inhibit lipid metabolism. This suggests that ECD can reduce body weight, abdominal circumference, and levels of TG and TC in HFD mice by activating and regulating the expression of PPARγ (Montaigne et al., 2021).

Therefore, ECD represents an effective combination of lipid-lowering metabolites that regulate apolipoproteins, apolipoprotein receptors, and lipid transport mechanisms.

### 3.2 Erchen Decoction modulates oxidative stress response

Oxidative stress arises from the excessive accumulation of reactive oxygen species generated during aerobic metabolism (Zhang S. et al., 2020). Increasing evidence suggests that oxidative stress can lead to cellular damage and death, contributing to conditions such as atherosclerosis, hyperlipidemia, stroke, and other cardiovascular diseases (Balan et al., 2024). It is also an important pathological factor for the development of atherosclerotic lesions. Studies have demonstrated that the combination of ECD and Taohong Siwu Decoction can enhance the expression of antioxidant factors, such as total superoxide dismutase (T-SOD) and glutathione (GSH), by regulating the p53/SLC7A11 signaling pathway. This combination reduces malondialdehyde (MDA) levels and alleviates oxidative stress in mice, showing significant effects on atheroma (He, 2022). Similarly, Zhang et al. reported that after treating mice with hyperlipidemic phlegm-turbidity obstructive syndrome with ECD, the transcription levels of intercellular adhesion molecule (ICAM) and E-selectin genes in the aortic endothelium of all treatment groups decreased, along with significant reductions in serum MDA concentrations. Following transcription, ICAM and E-selectin genes promote leukocyte adhesion to endothelial cells, facilitate the transformation of monocytes into macrophages, and contribute to the formation of lipid plaques, aggravating oxidative stress and leading to endothelial damage (Zhang et al., 2024). Therefore, ECD can reduce serum oxidative stress and decrease the transcription of aortic endothelial adhesion molecules, thereby playing a protective role in the vascular endothelium. It is speculated that this mechanism may be related to the downregulation of p38/MAPK signaling pathway activation.

### 3.3 Erchen Decoction inhibits the expression of inflammatory factors

Hyperlipidemia is closely associated with inflammatory responses that promote fat accumulation in the liver, thereby exacerbating inflammation and ultimately leading to elevated blood lipid levels, thereby creating a vicious cycle. Studies have shown that inflammatory factors are highly expressed in patients with hyperlipidemia and are positively correlated with TC, TG, and LDL-C (Shen, 2017). In a study conducted by Wang et al., it was observed that after treatment of ApoE−/−AS mice with ECD combined with Taohong Siwu Decoction, the expression levels of serum inflammatory factors TNF-α and IL-6 decreased, while the levels of nitric oxide (NO) and endothelin-1 (ET-1) increased significantly. Concurrently, the mRNA levels of transforming growth factor beta (TGF-β) and phosphoinositide 3-kinase (PI3K), as well as the protein phosphorylation levels of protein kinase B (AKT) and endothelial nitric oxide synthase (eNOS), were significantly reduced. It is speculated that ECD combined with Taohong Siwu Decoction may improve dyslipidemia and inflammatory responses by inhibiting the Nox4/NF-κB/HIF-1α signaling pathway, thus positively impacting the treatment of atherosclerosis (Wang et al., 2019). CRP is an acute-phase reactive protein produced by the liver cells that accelerates the inflammatory response *in vivo*. It is induced by tumor necrosis factor (TNF), IL-8, IL-6, and other inflammatory factors in mononuclear macrophages. Research has demonstrated that increased CRP levels can disrupt lipid metabolism and accelerate atherosclerosis (López-Mejías et al., 2016). Dong et al. reported that the application of ECD combined with statins significantly reduced the serum levels of IL-18, TNF-α, and high-sensitivity CRP (hs-CRP) in elderly patients with coronary heart disease and hyperlipidemia, suggesting a role in regulating dyslipidemia and suppressing inflammatory responses (Dong et al., 2021).

**FIGURE 2:**
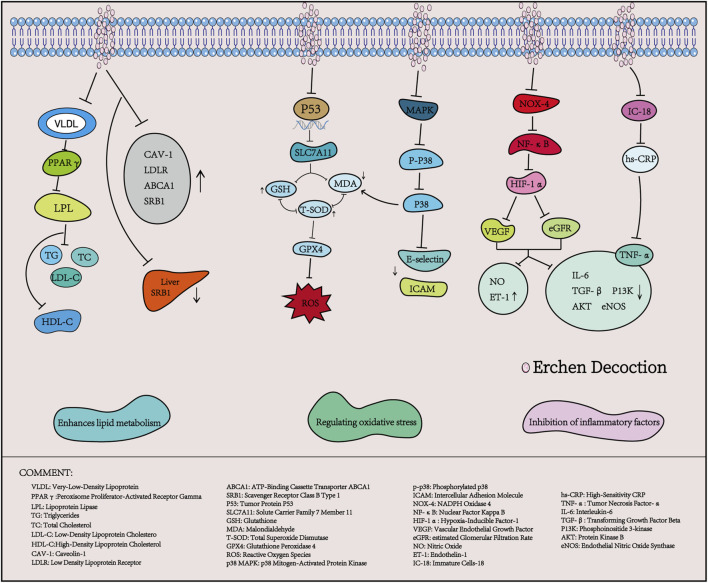
Mechanism of action of erchen decoction in treating hyperlipidemia.

In summary, ECD plays a significant role in the treatment of hyperlipidemia by regulating apolipoprotein processes, reducing oxidative stress responses, and inhibiting the expression of inflammatory factors (Table 3; Figure 2). Further research is needed to explore additional mechanisms of action.

**TABLE 3:** Research on the mechanism of Erchen Decoction.

Prescription composition (dosage of drug)	Effective concentrations	Real modules (animal/cell/patients)	Possible mechanisms	References	Apply styles
Pinellia ternata, tangerine peel, Poria cocos, l Glycyrrhiza uralensis (90g, 90g, 81g, 27g)	10 mL/kg/d	HFD-fed rats	Regulating the expression of Cav-1, LDLR, and ABCA1 mRNA in the liver	Ding et al. (2018)	*in vitro*
Pinellia ternata, tangerine peel, Poria cocos, glycyrrhiza uralensis (180g, 180g, 108g, 54g)	8.7 g/(kg· d) of ECD, simvastatin 2 mg/(kg·d)	C57BL/6 mice	Activating and affecting PPARγ expression	Zhang et al. (2020a)	*in vitro*
Pinellia ternata, tangerine peel, Poria cocos, Glycyrrhiza uralensis, Peach kernel, Safflower, Angelica, Chuanxiong, Red peony, Rehmannia (15g, 15g, 12g, 6g, 12g, 9g, 12g, 9g, 9g, 12g)	0.72, 1.44, 2.89 g/mL	SPF ApoE−/−mice,C57BL/6J mice	Inhibiting the Nox4/NF-κB/HIF-1α signaling pathway to improve dyslipidemia and inflammatory responses	Wang et al. (2019)	*in vitro*
Pinellia ternata, tangerine peel, Poria cocos, licoric, Codonopsis pilosula, Rhizoma Alismatis, radix bupleuri, hawthorn, Angelica sinensis, peach kernel, Safflower, Salvia miltiorrhiza, Radix Paeoniae Rubra, Ligusticum chuanxiong, Achyranthes bidentata (10g, 10g, 15g, 6g, 15g, 20g, 15g, 10g, 10g, 6g, 6g, 20g, 20g, 10g, 10g)		Elderly patients with coronary heart disease and hyperlipidemia	Regulating the levels of TC, TG, LDL-C, IL-18, TNF-α, and hs-CRP in elderly patients with coronary heart disease and hyperlipidemia	Luo (2014)	*in vivo*
prepared rehmannia root, Radix Paeoniae Rubra, Angelica sinensis ,chuanxiong rhizome, peach seed, Flos Carthami, tangerine peel, Pinellia ternata, Poria cocos, Glycyrrhiza uralensis (12g, 9g, 12g, 9g,12g, 9g, 15g, 15g, 12g, 6g)	0.72 g/mL, 1.44 g/mL, 2.89 g/mL	C57BL/6Jwild mouse, C57BL/6J background ApoE−/−mice	Regulating the p53/SLC7A11 signaling pathway to effectively inhibit oxidative damage and ferroptosis in EA.hy926 cells induced by ox-LDL.	He (2022)	*in vivo*
Pinellia ternata, tangerine peel, Poria cocos, Fructus mume, Zingiber, officinale, Glycyrrhiza uralensis (15g, 10g, 15g, 3g, 10g, 6g)	4.45g/(kg·d),8.90g/(kg·d),17.80g/(kg·d)	ApoE knockout mice, C57BL/6J mice	Downregulating the p38MAPK signaling pathway improves dyslipidemia and phlegm-turbidity obstruction syndrome by reducing the transcription levels of ICAM and E-Selectin genes and decreasing oxidative stress	Zhang et al. (2024)	*in vivo*

## 4 Pharmacological research on the six active metabolites in Erchen Decoction

### 4.1 Pinellia ternata

*Pinellia ternata*, a dried tuber from the Araceae family (Thunb), is characterized by its pungent and (Abo-Zaid et al., 2023)warm properties, along with its toxicity. It primarily targets the meridians of the spleen, stomach, and lung. Its therapeutic functions include drying dampness and transformation of phlegm. Clinically, it is commonly used to treat cough, asthma, nausea, vomiting, and scrofula. Modern pharmacological studies have shown that P. ternata possesses anti-inflammatory, antibacterial, and lipid-lowering effects (Zou et al., 2023). In TCM, it is recognized for its efficacy in alleviating vomiting and reducing lumps and distension (Geng et al., 2023). *Pinellia ternata* contains a variety of metabolites, including alkaloids, organic acids, amino acids, phenylpropanoids, and volatile oils (Wang and Wang, 2020). The Alkaloids include various components such as ephedrine, indole alkaloids, isoquinoline alkaloids, and purine alkaloids (Dey et al., 2020), Which Organic acids can be classified into fatty acids, saturated or unsaturated acids, and acids with different numbers of carboxyl groups (Shi et al., 2022).

As the monarch drug in the metabolite formula, P. ternata can directly reduce the expression of TG, LDL-C, IL-6, TNF-α, and other inflammatory markers by mediating the PI3K/Akt pathway, resulting in improved blood lipid levels (Lu et al., 2020). Processed P. ternata has been shown to effectively lower TG and LDL-C levels, improve whole blood viscosity, and inhibit red blood cell aggregation (Yin and Fu, 2022b).

Phytosterols, which are structurally similar to cholesterol, compete with cholesterol for incorporation into micelles, thereby inhibiting their absorption and affecting the synthesis of endogenous cholesterol. This mechanism helps regulate fat formation and reduce circulating triglyceride levels (Lu et al., 2019). *Pinellia ternata* is rich in phytosterols, including stigmasterol and β-sitosterol, both of which exhibit anti-obesity and lipid-lowering effects. Xin et al. found that stigmasterol significantly reduced liver cholesterol levels in mice fed a high-fat, high-cholesterol diet, inhibited the expression of nucleotide-binding oligomerization domain-like receptor protein 3 (NLRP3) inflammasome and IL-18 genes (*p* < 0.05), lowered TG and TC levels in the liver, and enhanced the α-alternative pathway of the intestinal microbiota (Xin et al., 2023). Zhang et al. also discovered that β-sitosterol can reverse the intestinal microbiome imbalance in mice fed an HFD, modulate bile acid metabolism through the intestinal microbiome and CYP7A1 pathway, effectively alleviating metabolic disorders in mice and exhibit therapeutic effects on obesity and hyperlipidemia induced by HFD (Zhang et al., 2022).

Furthermore, studies have shown that β-sitosterol can decrease serum LDL-C levels in mice, alter lipid profiles, and achieve effects comparable to conventional lipid-lowering medications (Zhang et al., 2024). A study by Abo-Zaid et al. demonstrated that β-sitosterol treatment significantly improved hyperglycemia, transaminase levels (ALT and AST), and lipid levels in HFD rats, suggesting that β-sitosterol directly treats NAFLD by regulating lipid metabolism and alleviating endoplasmic reticulum stress, oxidative stress, and inflammatory responses (Abo-Zaid et al., 2023). Research by Gumede et al. indicated that β-sitosterol effectively prevented NAFLD and large vacuolar fatty change in rats fed a high-fructose diet (Gumede et al., 2020). In conclusion, both stigmasterol and β-sitosterol can improve triglyceride and cholesterol levels, reduce intestinal bile acid levels, and enhance the expression of genes involved in lipid metabolism, thereby ameliorating hyperlipidemia (Feng et al., 2018).

### 4.2 Tangerine peel

Tangerine peel is characterized by bitter, spicy, and warm properties, targeting the spleen and lung meridians. Its therapeutic effects include regulation of qi, strengthening of the spleen, drying dampness, and elimination of phlegm. The main metabolites of tangerine peel can be categorized into volatile and non-volatile substances. The volatile metabolites comprise over 300 metabolites, including ketones, alcohols, terpenes, acids, phenols, and ethers, whereas the non-volatile metabolites include more than a hundred types, such as flavonoids, alkaloids, and triterpenes. Flavonoids are the most significant metabolites in tangerine peel (Guan et al., 2024). Studies have shown that Citrus flavonoids can significantly inhibit dyslipidemia in conditions such as hepatic steatosis and obesity by reducing inflammatory responses associated with tissue metabolism in the liver, adipose tissue, and kidneys (Mulvihill et al., 2016). In animal experiments, Burke’s research team was able to reverse the size and quantity of existing fat, reduce plasma cholesterol, and improve hyperlipidemia by enhancing fatty acid oxidation in the liver and increasing the overall energy expenditure (Burke et al., 2018).

Nobiletin, a polymethoxylated flavonoid derived from citrus peels, exhibits potent anti-inflammatory and antioxidant properties. It primarily prevents hepatic steatosis by enhancing fatty acid oxidation and inhibiting hepatic fatty acid synthesis, thereby playing a crucial role in reducing lipid abnormalities and alleviating atherosclerosis (Mulvihill et al., 2016). Research conducted by Prapassorn et al. found that upregulating the Nrf-2/HO-1 signaling pathway and inhibiting matrix metalloproteinases (MMPs) can alleviate vascular remodeling and functional disorders in L-NAME-induced HFD rats (Potue et al., 2019). In another study, Bunbupha et al. demonstrated that nobiletin could moderate the effects of HFD on the expression of liver adiponectin receptor 1 (AdipoR1) and gp91^phox^, thereby regulating adiponectin levels, reducing oxidative stress, and alleviating metabolic disorders in rats after high-fat intake (Bunbupha et al., 2021). Additionally, Kim et al. found that continuous supplementation of HFD rats with low doses of nobiletin over 16 weeks resulted in reduced plasma total cholesterol, apolipoprotein B (ApoB), and non-high-density lipoprotein levels, ultimately improving dyslipidemia (Kim et al., 2017).

*Pinellia ternata* and tangerine peel form a high-frequency drug pair for hyperlipidemia treatment. Together, they effectively address arterial endothelial thickening and can enhance the levels of reactive oxygen species in cells through the PI3K-Akt pathway, controlling cell apoptosis and delaying aging (Sun et al., 2018). Zhang et al. investigated the mechanisms of hyperlipidemia via multi-pathway and multi-target interactions of the active metabolites in tangerine peel and P. ternata using network pharmacology, and discovered that this combination significantly improved blood lipid profiles in hyperlipidemic mice (Zhang, 2022).

### 4.3 Poria cocos (fu ling)

*Poria cocos*, which is characterized by its sweet, light, and mild properties, targets the heart, lungs, spleen, and kidney meridians. Its therapeutic effects include promoting diuresis, relieving dampness, strengthening the spleen, and tranquilizing the mind and spirit. *Poria cocos* primarily contains various metabolites, including polysaccharides, sterols, triterpenes, proteins, amino acids, organic acids, esters, flavonoids, and trace elements, with polysaccharides being the most abundant (Lü et al., 2024).

*Poria cocos* polysaccharides (PCP) are among the main active and characteristic metabolites of P. cocos, comprising approximately 80% of the bioactive metabolites in its sclerotium. PCP exhibits anti-inflammatory and antioxidant properties (Ll, 2016; Cheng et al., 2020). β-Glucans are the predominant metabolites of PCP and are characterized by β-(1→3) glucan backbones and β-(1→6) glucan side chains (Li X. et al., 2019). Li et al. showed that PCP can inhibit inflammatory response factors induced by HFD in ApoE^−/−^ mice by controlling the elevation of TNF-α, IL-6, and nitric oxide, inhibiting the activation of the aortic TLR4/NF-κB pathway, and reducing lipid accumulation (Li et al., 2021). Wei et al. administered low, medium, and high doses of PCP to nutritionally obese rats, found that medium and high doses significantly decreased the serum levels of TC, TG, and LDL-C, indicating that PCP can effectively reduce serum lipid levels and regulate lipid abnormalities, demonstrating its lipid-lowering effects (Wei et al., 2023).

*Poria cocos* oligosaccharides are derived from PCP through processes such as enzymatic hydrolysis and purification, resulting in improved water solubility compared to that of the original polysaccharides. These oligosaccharides can inhibit metabolic disorders in mice fed an HFD, reduce inflammatory responses, and decrease the accumulation of lipid abnormalities (Zhu et al., 2022a). Furthermore, Zhu et al. used P. cocos oligosaccharides to reshape the gut microbiota structure in mice, inhibit intestinal barrier damage, repair insulin resistance and glucose tolerance, and improve the dysregulation of glucose and lipid metabolism (Zhu et al., 2022b).

### 4.4 Glycyrrhiza uralensis

*Glycyrrhiza uralensis*, commonly known as licorice, is sweet and mild in properties, and targets the heart, lung, spleen, and stomach meridians. Its therapeutic effects include tonifying the spleen and supplementing qi, clearing heat detoxifying, expelling phlegm, relieving cough, alleviating acute pain, and harmonizing various drugs. The chemical metabolites of G. uralensis mainly consist of triterpenoids such as glycyrrhizin, glycyrrhetinic acid, and glycyrrhizic acid; flavonoid metabolites including liquiritin, isoliquiritin, glycyrrhizin, and isoliquiritigenin; as well as licorice polysaccharides, coumarins, alkaloids, amino acids, and a small amount of volatile metabolites (Xiao et al., 2023).

Pan et al. extracted polysaccharides from medicinal G. uralensis and conducted a series of analyses, leading to the development of a new type of Glycyrrhiza inflata batalin polysaccharide (GIBP). Its antioxidant and anti-α-glucosidase properties effectively alleviate hyperglycemia (Pan et al., 2020). Wu et al. employed the reverse evaporation method to prepare Glycyrrhiza polysaccharide liposomes (GPSL) and optimized them. The results indicated that both GPSL and Glycyrrhiza polysaccharide (GPS) had immunomodulatory effects on chBM-DCs, with GPSL showing a more significant effect than GPS (Wu et al., 2017). Wu et al. found that after administering Glycyrrhiza polysaccharides to mice via gavage, pro-inflammatory cytokines such as IL-6, interleukin-7, interleukin-10, and TNF-α, were reduced, and the antioxidant capacity was significantly enhanced (Wu et al., 2020). Cao et al. extracted glycyrrhetinic acid (GA) from licorice and discover its anti-inflammatory properties and ability to mediate the NF-κB pathway, inhibiting the expression of downstream inflammatory factors TNF-α, IL-1β, IL-6, and IL-8, thus reducing cytotoxicity (Cao et al., 2017). Wang et al. confirmed that after administering high and low doses of GPS to mice via gavage, the high-dose group showed significant reductions in TC, TG, and LDL-C levels, while HDL-C levels were elevated, in contrast to the low-dose group. This suggests that GPS participates in lipid metabolism in type 1 diabetes mellitus (T1DM) mice, regulating lipid metabolism and improving dyslipidemia (Wang et al., 2023).

Additionally, other metabolites of G. uralensis have therapeutic or alleviating effects on hyperlipidemia. Carbenoxolone, an active metabolite in licorice, has demonstrated potential in treating obesity and hyperlipidemia by activating the JAK2/STAT3 signaling pathway and reducing the expression of sterol regulatory element-binding protein 1c (SREBP-1c) and fatty acid synthase (FAS), thus protecting the liver from lipid metabolic damage induced by a HFD (Chen et al., 2019). GA, another flavonoid metabolite from G. uralensis, was used by Weng et al. in experiments with a hyperlipidemic mouse model, which it alleviated lipid metabolism disorders and enhanced the lipid-lowering effects of dioscin stem cells (Weng et al., 2021).

### 4.5 Zingiber officinale

Zingiber officinale (Z. officinale), commonly known as ginger, is characterized by its spicy and slightly warm properties, targeting the lung, spleen, and stomach meridians. It exerts effects such as dispersing the exterior cold, warming the middle to stop vomiting, eliminating phlegm, relieving cough, and detoxifying fish and crab toxins. *Zingiber officinale* contains over 200 metabolites, predominantly volatile oils and amino acids. Volatile oils comprise metabolites such as α-zingiberene, β-sesquiphellandrene, shogaol-3, shogaol-4, gingerol, and zingerone, including aspartic acid, glutamic acid, and serine, among others (Wu et al., 2019).

Cheng et al. induced hyperlipidemia in mice using a HFD and discovered that 6-shogaol inhibited hypertrophy and hyperplasia of white adipose tissue (WAT) in mice, down-regulating the TLR3/IL-6/JAK1/STAT3 and PCNA signaling axes, thereby improving liver metabolic disorders and insulin resistance (Cheng et al., 2022). Supplementation with steamed ginger ethanolic extract (SGE) reduces SREBP1c (Srebf1), a factor that promotes lipogenesis, leading to weight loss and decreased body fat in mice. Furthermore, SGE inhibited fat formation and accumulation by lowering key regulators of adipogenesis such as Pparg and Cebpa. Dietary control combined with moderate exercise and supplementation with SGE further supports weight and fat reduction (Park et al., 2020). Suk et al. treated HFD mice with gingerenone A (GA) and found that GA inhibited adipocyte hyperplasia and macrophage infiltration, alleviated symptoms associated with adipose tissue, reduced adipose tissue inflammation (ATI), and the occurrence of obesity (Suk et al., 2017). Gingerol, which is present in the rhizomes of Z. officinale, was studied by Olivarez et al., who found that a mixture of 6-gingerol, 8-gingerol, and 10-gingerol exhibited anti-lipogenic and lipolytic effects on the 3T3-L1 cell line, confirming the anti-obesity effects (Gembe-Olivarez et al., 2023).

### 4.6 Fructus mume

Fructus mume (F. mume), commonly known as Mume Fructus or dark plum, is characterized by its sour, astringent, and mild properties with channel tropism, including the liver, spleen, lung, and large intestine meridians. It exerts effects such as astringing the lungs, binding the intestines, generating fluids, and calming roundworms (Zhu Y. et al., 2022). The main metabolites of F. mume include terpenoids, organic acids, polysaccharides, amino acids, volatile components, nucleotides, and inorganic elements (Ou et al., 2020). The triterpene metabolites in F. mume primarily include oleanolic acid (OA) and ursolic acid (UA) (Bailly, 2020).

OA, a pentacyclic triterpene metabolite, has been shown to lower blood lipids, demonstrates anticancer and anti-inflammatory properties, and prevents cardiovascular and cerebrovascular diseases (Shang and Bai, 2022). Research indicates that OA can improve glucose tolerance and visceral fat in mice, thereby regulating fat and carbohydrate metabolism and intervening in hyperlipidemia (de Melo et al., 2010). Hepatocyte nuclear factor 1b (HNF1b) is a crucial regulator of lipid and glucose metabolism, and is capable of regulating obesity and redox homeostasis (Wang et al., 2017). OA can regulate and inhibit oxidative damage and glucose/lipid metabolic dysfunction induced by polychlorinated biphenyl mixtures via HNF1b-mediated redox homeostasis and PPARγ signal transduction (Su et al., 2018). Luo et al. administered OA to patients with hyperlipidemia for 4 weeks, which resulted in significant decreases in serum TC and TG levels, and an increase in HDL-C. DNA microarray results also indicated significant changes in mRNA expression; expression increased in 17 patients and decreased in four patients post-treatment, providing evidence for the effectiveness of OA in improving hyperlipidemia (Luo et al., 2018).

UA is a natural triterpenoid carboxylic acid metabolite. Previous studies have demonstrated that UA has anti-inflammatory, antioxidant (Zhao et al., 2023), and lipid-lowering effects (Ruan et al., 2019). Ma et al. found that UA could regulate MAPK and NF-κB pathways by inhibiting the expression of TNF-α, IL-1, and COX-2 proteins, thereby reducing liver oxidative stress and inflammation induced by carbon tetrachloride (CCl4) in mice (Ma et al., 2014). Ruan et al. fed rats different concentrations of UA and found that it effectively reduced lipid synthesis, leading to decreased levels of TG, TC, and LDL (Ruan et al., 2019). Li et al. reported that by feeding atherosclerosis mice UA and rosuvastatin, there was a significant reduction in the necrotic core area in blood vessels, a decrease in atherosclerotic plaque area, and inhibition of NF-κB-mediated LOX-1 expression *in vivo* and *in vitro* through ROS production, which improved outcomes related to atherosclerosis (Li et al., 2018).

**FIGURE 3:**
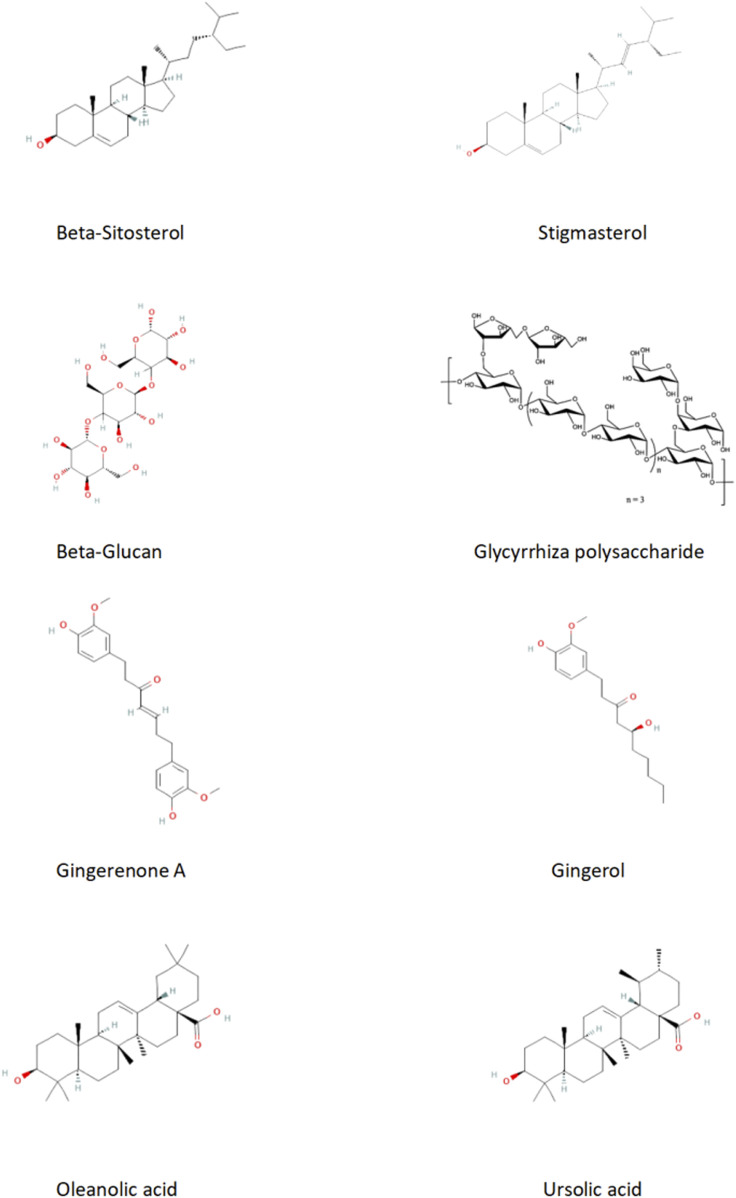
Effective chemical components of erchen decoction in the treatment of hyperlipidemia.

In summary, the effective chemical metabolites of the ECD in treating hyperlipidemia primarily include β-sitosterol, nobiletin, PCP, PCP, licorice polysaccharides, GA, gingerol, OA, UA. These metabolites exhibit therapeutic effects on hyperlipidemia through different pathways and mechanisms (Table 4; Figure 3). However, the specific chemical metabolites of ECD contributing to its effects on hyperlipidemia remain to be clearly defined and warrant further investigation by future researchers.

**TABLE 4:** Active metabolites and their functions in Erchen Decoction.

Chemical metabolites	Source plant	Main functions	Molecular formula	Molecular weight	CAS number	Related research
β-sitosterol	*Pinellia ternata*	Lowering blood lipids, anti-obesity	C29H50O	414.72 g/mol	83-46-5	Xin et al. (2023), Zhang et al. (2024)
Nobiletin	*Tangerine Peel*	Anti-inflammatory, antioxidant, improving lipid abnormalities	C21H22O8	402.4 g/mol	478-01-3	Mulvihill et al. (2016), Kim et al. (2017)
β-Glucan	*Poria cocos*	Anti-inflammatory, antioxidant, lowering blood lipids	C18H32O16	504.4 g/mol	9041-22-9	Li et al. (2021), Wei et al. (2023)
Glycyrrhiza polysaccharide	*Licorice*	Regulating lipid metabolism, improving hyperlipidemia	-	-	9000-45-7	Wang et al. (2023), Wu et al. (2020)
Gingerenone A	*Ginger*	Anti-obesity, improving lipid metabolism	C21H24O5	356.4 g/mol	128,700-97-0	Suk et al. (2017)
Gingerol	*Ginger*	Anti-lipogenesis,improving metabolism	C17H26O4	294.4 g/mol	23,513-14-6	Gembe-Olivarez et al. (2023)
Oleanolic acid	*Dark Plum*	Lowering blood lipids,anti-inflammatory, improving metabolism	C30H48O3	456.7 g/mol	508-02-1	Luo et al. (2018), Shang and Bai (2022)
Ursolic acid	*Dark Plum*	Anti-inflammatory,antioxidant, lowering blood lipids	C30H48O3	456.71 g/mol	77-52-1	Ruan et al. (2019), Ma et al. (2014)

## 5 Discussion

Hyperlipidemia is closely related to factors such as age, diet, exercise, and genetics (Opoku et al., 2021; Trinder et al., 2022). In its early stages, hyperlipidemia often presents no obvious symptoms, making it easy to overlook. It is typically discovered during routine check-ups or when other diseases arise, which can lead to serious consequences (Libby et al., 2019). Statins are notably effective in treating hyperlipidemia and significantly reduce mortality rates (Cai et al., 2021). However, owing to their long-term use and potential side effects, TCM has been actively seeking alternative treatments.

ECD, which is known for its ability to dispel phlegm and dampness, has long been effective in treating pulmonary diseases (Deng et al., 2020; Ye et al., 2023). Recently, its application in the treatment of hyperlipidemia has increased, indicating a broader scope for research (Chen et al., 2023). Through a literature review, it has been found that current studies on ECD for hyperlipidemia primarily focus on clinical observations, the mechanisms of metabolite action, the molecular mechanisms of individual botanical drugs, pharmacological effects, and the study of chemical metabolites. Although existing research has sufficiently demonstrated the efficacy of ECD, there are still notable shortcomings. First, there are significant discrepancies in the timing and length of clinical and experimental studies, and there is a lack of international literature outside of China. Second, as this paper is a review, patient-level data cannot be obtained, which limits the in-depth analysis of clinical effects. Lastly, current research on effective metabolites mainly concentrates on individual botanical drugs, while the effective metabolites of ECD in treating hyperlipidemia remain underexplored. The interactions among individual botanical drugs during the decoction process may influence the overall efficacy, and further research is needed to evaluate the efficacy and toxicity, as well as the principles of botanical drug combinations.

Therefore, future research should primarily focus on the effective metabolites of ECD, particularly the active metabolites of the metabolite and their interactions, to elucidate the mechanisms by which it treats hyperlipidemia. Additionally, subsequent studies should comprehensively assess the efficacy and safety of ECD through *in vivo*, *in vitro*, and cellular experiments to ensure its effectiveness and minimal side effects in clinical applications. Attention should also be paid to drug toxicity and the principles of botanical drug combinations to optimize treatment plans and provide theoretical guidance.

A total of 39 articles were sourced from China. While it is challenging to obtain international qualifications, all formulas and traditional Chinese medicines have a rich historical background and comply with the standards set by the Chinese Pharmacopoeia (2015). Therefore, we standardized the relevant Chinese literature using an international taxonomy website in conjunction with the Chinese Pharmacopoeia (2015) (Supplementary Appendix S1). Based on the standards established by the ConPhyMP Declaration (Heinrich et al., 2022), we conducted a detailed assessment of chemical metabolites in the articles (Supplementary Appendix 2A).

In summary, ECD demonstrates significant efficacy in clinical applications for the treatment of hyperlipidemia. In animal experiments, it has been shown to act on hyperlipidemia by enhancing lipid metabolism, regulating oxidative stress responses, and inhibiting the expression of inflammatory factors. Research on the individual effective metabolites in treating hyperlipidemia is clear, and future studies should continue to refine research methodologies that integrate basic practice with experimental approaches, providing more references for the prevention and treatment of hyperlipidemia.

## Abbreviations

ECD, Erchen Decoction; TC, total cholesterol; TG, triglycerides; LDL-C, low-density lipoprotein cholesterol; HDL-C, high-density lipoprotein cholesterol; ASCVD, atherosclerotic cardiovascular diseases; CVD, Cardiovascular diseases; NAFLD, non-alcoholic fatty liver disease; Pinellia ternate, P. ternate; Poria cocos, P. cocos; Glycyrrhiza uralensis, G. uralensis; Zingiber officinale, Z. officinale; Fructus mume, F. mume; TNF-α, Tumor Necrosis Factor-α; CRP, C-reactive protein; TNF, tumor necrosis factor; IL-6, Interleukin-6; HFD, high-fat diet; IL-8, Interleukin-8; hs-CRP, high-sensitivity C-reactive protein; PCP, Poria cocos Polysaccharides; GIBP, Glycyrrhiza inflata Batalin polysaccharide; GPSL, Glycyrrhiza polysaccharide liposome; GA, glycyrrhetinic acid; OA, oleanolic acid; HNF1b, Hepatocyte nuclear factor 1b; UA, Ursolic acid.
